# Are two readers more reliable than one? A study of upper neck ligament scoring on magnetic resonance images

**DOI:** 10.1186/1471-2342-13-4

**Published:** 2013-01-17

**Authors:** Ansgar Espeland, Nils Vetti, Jostein Kråkenes

**Affiliations:** 1 Department of Radiology, Haukeland University Hospital, Jonas Liesvei 65, 5021, Bergen, Norway; 2 Department of Surgical Sciences, University of Bergen, Bergen, Norway

## Abstract

**Background:**

Magnetic resonance imaging (MRI) studies typically employ either a single expert or multiple readers in collaboration to evaluate (read) the image results. However, no study has examined whether evaluations from multiple readers provide more reliable results than a single reader. We examined whether consistency in image interpretation by a single expert might be equal to the consistency of combined readings, defined as independent interpretations by two readers, where cases of disagreement were reconciled by consensus.

**Methods:**

One expert neuroradiologist and one trained radiology resident independently evaluated 102 MRIs of the upper neck. The signal intensities of the alar and transverse ligaments were scored 0, 1, 2, or 3. Disagreements were resolved by consensus. They repeated the grading process after 3–8 months (second evaluation). We used kappa statistics and intraclass correlation coefficients (ICCs) to assess agreement between the initial and second evaluations for each radiologist and for combined determinations. Disagreements on score prevalence were evaluated with McNemar’s test.

**Results:**

Higher consistency between the initial and second evaluations was obtained with the combined readings than with individual readings for signal intensity scores of ligaments on both the right and left sides of the spine. The weighted kappa ranges were 0.65-0.71 vs. 0.48-0.62 for combined vs. individual scoring, respectively. The combined scores also showed better agreement between evaluations than individual scores for the presence of grade 2–3 signal intensities on any side in a given subject (unweighted kappa 0.69-0.74 vs. 0.52-0.63, respectively). Disagreement between the initial and second evaluations on the prevalence of grades 2–3 was less marked for combined scores than for individual scores (*P* ≥ 0.039 vs. *P* ≤ 0.004, respectively). ICCs indicated a more reliable sum score per patient for combined scores (0.74) and both readers’ average scores (0.78) than for individual scores (0.55-0.69).

**Conclusions:**

This study was the first to provide empirical support for the principle that an additional reader can improve the reproducibility of MRI interpretations compared to one expert alone. Furthermore, even a moderately experienced second reader improved the reliability compared to a single expert reader. The implications of this for clinical work require further study.

## Background

A key feature of any imaging test is its reliability [1]. A conclusive image reading should be reliable, regardless of the number of readers involved. Higher reliability might be expected when multiple readers interpret the images and their readings are combined than when only one reader interprets the images. However, we have found no empirical data to support or refute this assumption. A majority or consensus view is not necessarily more reliable than the view of one expert alone. Two readers combined could, in theory, be less consistent than one reader (as exemplified by the hypothetical data shown in Additional file 1). Double, or repeated readings can also affect validity; this approach can prevent errors [2,3], but it can also increase false positive rates [4].

Radiologists provide a large number of expert opinions in their daily work. This work could be significantly impacted by data that showed a single expert opinion was insufficient or that a second opinion provided additional benefit. In research settings, it is more feasible for a single expert to study large numbers of images, rather than multiple readers. Indeed, many studies have reported conclusive image findings based on the determination of only one expert reader [5-7]. In other studies, multiple readers were used to determine the final image results [8-11]. We compared these two approaches for scoring the signal intensities of the alar and transverse ligaments on upper neck magnetic resonance images (MRIs). Consistent image readings are required in research to assess the presence and clinical relevance of high intensity signals [11-13]. Our aim was to determine whether consistency in image interpretation by a single expert might be equal to the consistency of combined readings, defined as independent interpretations by two readers, where cases of disagreement were reconciled by consensus.

## Methods

This study included 102 prospectively recruited subjects (49 men and 53 women; mean age 47.2 years) that comprised 68 healthy volunteers, 18 patients with rheumatoid arthritis, and 16 patients with chronic neck pain. These subjects represented random subsamples of participants included in a larger project on MRIs of upper neck ligaments. Based on a computer generated list of random numbers, the present study included the same relative numbers of healthy individuals, patients with arthritis, and patients with neck pain as were included in the larger project. All subjects gave written informed consent to participate. The study was in compliance with the Helsinki Declaration and was approved by The Regional Committee for Medical Research Ethics, Western-Norway.

All subjects were imaged with their head and neck in a neutral position in a standard, one-channel, circular, polarized, receive-only, head coil, with a 1.5 Tesla scanner (Symphony Mastroclass, Siemens Medical System, Erlangen, Germany). We used an established protocol for MRI of upper neck ligaments [14]. This protocol included proton-density-weighted fast-spin echo sequences of the upper neck in the axial, coronal, and sagittal planes with the following parameters: repetition time: 2150–2660 ms, echo time: 15 ms, slice thickness: 1.5 mm, interslice gap: 0.0 mm or 0.3 mm (sagittal), field of view: 175 mm × 200 mm or 200 mm × 200 mm (coronal), voxel size: 0.6-0.7 × 0.4 × 1.5 mm^3^, and echo train length: 13.

The alar and transverse ligaments were scored on a scale of 0, 1, 2, or 3 based on the ratio of the largest cross-sectional area of a high intensity signal (observed in at least two imaging planes) to the total cross-sectional area of the ligament [15,16]. A high intensity signal in 1/3 or less of the total cross sectional area was scored 1; a high intensity signal in 1/3 to 2/3 of the total cross sectional area was scored 2; and a high intensity signal in 2/3 or more of the total cross sectional area was scored 3. Homogenous grey ligaments were scored 2. Ligaments with no high intensity signal were scored 0. The right and left sides of the spine were scored separately; alar ligaments were scored on sagittal sections, and transverse ligaments were scored on sagittal or coronal sections, depending on ligament orientation.

One neuroradiologist (reader A) with 26 years experience and one radiology resident (reader B) with 6 years experience independently scored the signal intensities of the ligaments. Then, all disagreements were resolved by consensus. This process resulted in individual scores for each reader and combined scores for both readers (based on independent readings followed by consensus reading in cases of disagreement). Prior to this study, both readers were trained in the scoring system used and had discussed scores in joint meetings. Reader A had previously scored several thousand ligaments and reader B had scored about one thousand ligaments.

The images were de-identified, presented in a random order (according to a computer generated list of random numbers), and interspersed among similar images that were not used in this study. After 3–8 months, the same images were presented in a new random order (according to a new list of random numbers), and again, interspersed among similar images. The readers were not told that they had assessed the images previously. Readers A and B independently re-scored the signal intensities of the ligaments and resolved any disagreements by consensus. Thus, they repeated the entire process followed in the first evaluations.

Agreements between the initial and second evaluations were analyzed for each reader and for the combined determinations. We analyzed evaluations of each of the four ligament parts separately (left and right alar ligaments, left and right parts of the transverse ligament) and all the ligament parts combined. We calculated linearly weighted kappa values to assess agreement on scores 0–3 for each side. We used unweighted kappa values to assess agreement on scores 2–3 vs. scores 0–1 per subject on any side (right and/or left). Kappa values are expressed with 95% confidence intervals (CIs) based on SEs (non-zero) and were interpreted as follows: k ≤ 0.20, poor; 0.21-0.40, fair; 0.41-0.60, moderate; 0.61-0.80, good; and 0.81-1.00, very good agreement beyond chance [17]. Disagreements on the prevalence of scores 2–3 were assessed with McNemar’s test. *P* < 0.05 was taken to indicate statistical significance.

We compared the sum of the scores for all four ligament parts (MRI sum score, 0–12) between reader A vs. reader B vs. their combined scoring vs. their average scoring regarding a) intra-reader reliability using intraclass correlation coefficients (ICCs, two-way model, assuming normality), b) smallest detectable change (SDC), and c) difference between the second and first evaluation using Bland Altman plots with 95% limits of agreement. ICC ≥ 0.70 suggested adequate reliability [18]. SDC was defined as 1.96×√2SEM (standard error of measurement) and indicated the smallest change in MRI sum score that, with *P* < 0.05, could be interpreted as a “real change” above measurement error in one individual [18]. Data were analyzed using WINPEPI 10.0 (http://www.brixtonhealth.com/pepi4windows.html).

A statistical power assessment indicated that, with a true, unweighted kappa of 0.70 and a prevalence of 30% for the relevant MRI finding (e.g., intensity signal scores of 2–3), 85 paired observations would provide 80% power to give a significant result at the 5% level in a two-sided test of k = 0.40 [19]. With a true, unweighted kappa of 0.60 and a prevalence of 30%, 191 paired observations would provide 80% power to give a significant result. This study included 102 paired observations, or 408 paired observations, including all ligament parts.

## Results

**Figure 1:**
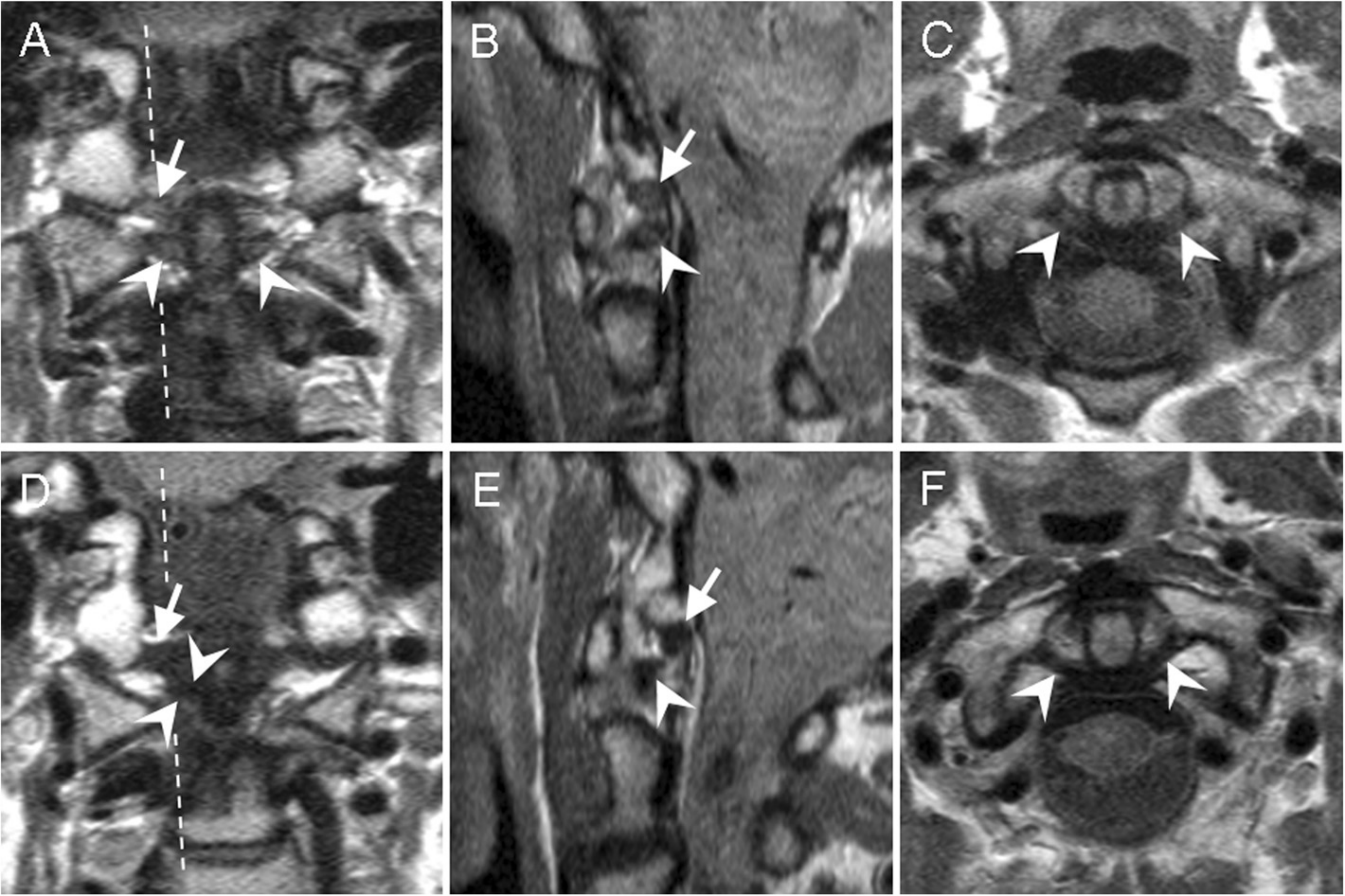
**Scoring high signal intensities of alar and transverse ligaments on upper neck MRIs.** Proton-density-weighted, fast-spin echo, 1.5 Tesla MRI sections were performed in **(A, D)** coronal, **(B, E)** sagittal, and **(C, F)** axial directions. MRIs were from two healthy women, aged **(A-C)** 44 years old, and **(D-F)** 60 years old. Broken lines mark the sagittal plane. **(A-C)** The transverse ligament is indicated with arrow heads. The high intensity signal was scored 2 by reader A, 1 by reader B, and 2 by consensus; in the second evaluation, the same signal was scored 2 by both readers independently. The alar ligament is indicated with arrows. **(A, B)** The high intensity signal was graded 2 by both readers independently; in the second evaluation, the same signal was scored 2 by reader A, 3 by reader B, and 2 by consensus. **(D-F)** The transverse ligament (arrow heads) and alar ligament (arrows) were scored 0 by both readers independently in both evaluations.

Better agreement between the initial and second evaluations of alar and transverse ligament signal intensities was obtained with the combined readings than with the individual readings (Table 1, Figure 1). This applied to either ligament side (weighted kappa range was 0.65-0.71 for combined readings vs. 0.48-0.62 for individual readings) and to the presence of grade 2–3 signal intensities on any ligament side in a given subject (unweighted kappa range was 0.69-0.74 for combined readings vs. 0.52-0.63 for individual readings) (Table 1). For all ligament parts combined (n = 408), the weighted kappas for agreement between the initial and the second evaluations were 0.56 (95% CI: 0.50, 0.62) for reader A, 0.55 (95% CI: 0.48, 0.62) for reader B, and 0.68 (95% CI: 0.63, 0.74) for the combined reading.

**Table 1:** Kappa values for agreement between initial and second evaluations

	**Alar ligament scores 0-3**			**Transverse ligament scores 0-3**		
**Scored by**	**Right side**	**Left side**	**Any side, scores 2-3**	**Right side**	**Left side**	**Any side, scores 2-3**
Reader A	0.59 (0.47, 0.70)	0.62 (0.51, 0.73)	0.63 (0.48, 0.78)	0.50 (0.35, 0.64)	0.51 (0.38, 0.64)	0.59 (0.43, 0.75)
Reader B	0.51 (0.39, 0.63)	0.57 (0.44, 0.70)	0.52 (0.35, 0.68)	0.48 (0.32, 0.64)	0.58 (0.45, 0.72)	0.53 (0.38, 0.68)
A and B combined	0.68 (0.56, 0.79)	0.71 (0.61, 0.81)	0.74 (0.61, 0.88)	0.66 (0.53, 0.79)	0.65 (0.54, 0.77)	0.69 (0.54, 0.84)

Values represent linearly weighted kappa values for scores 0, 1, 2, or 3 on each side of the spine, and unweighted kappa values for scores 2–3 vs. scores 0–1 per subject on any side (right and/or left), with 95% confidence intervals in parenthesis, based on magnetic resonance imaging in 102 subjects.

A higher prevalence of signal intensity scores of 2–3 per subject was reported in the second evaluations compared to the initial evaluations (Table 2). The *P* values for the difference between evaluations were smaller for individual reader scoring than for combined scoring (*P* ≤ 0.004 vs. *P* ≥ 0.039 for individual vs. combined differences, respectively) (Table 2).

**Table 2:** Prevalence of scores 2–3 on initial and second evaluations

	Alar ligament scores 2-3^*^			Transverse ligament scores 2-3^*^		
**Scored by**	**Initial %**	**Second %**	***P *value**^**§**^	**Initial %**	**Second %**	***P *value**^**§**^
Reader A	29.4	43.1	0.001	27.5	40.2	0.004
Reader B	22.5	40.2	<0.001	25.5	46.1	<0.001
A and B combined	31.4	39.2	0.039	28.4	36.3	0.057

The data are based on magnetic resonance imaging in 102 subjects.

^*^ Highest assigned score when different intensities were noted on the right and left sides of the spine.

^§^*P* value for difference in prevalence (%), based on McNemar’s test.

In the initial evaluation, the combined scores agreed with reader A’s scores in 86.0% of cases and with reader B’s scores in 80.1% of cases. In the second evaluation, the combined scores agreed with reader A’s scores in 83.3% of cases and with reader B’s scores in 77.7% of cases. Weighted kappa values for agreement between A and B was 0.49 (95% CI: 0.41, 0.57) in the initial evaluation and 0.56 (95% CI: 0.49, 0.62) in the second evaluation (all ligament parts combined, n = 408).

**Figure 2:**
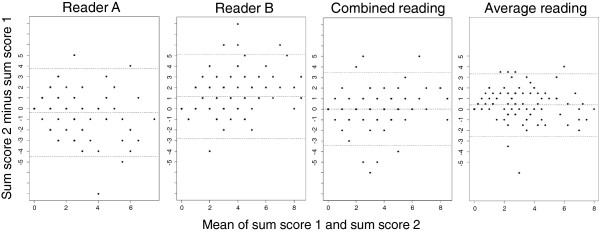
**Bland Altman plots of difference in MRI sum score between second and first evaluations.** On 102 upper neck MRIs, two readers A and B independently graded the signal intensities of the alar and transverse ligaments on both sides of the spine (i.e. four ligament parts) on a scale of 0, 1, 2 or 3 and then resolved disagreements by consensus (combined reading). They also repeated the grading process (second evaluation). The sum of the scores for all ligament parts (MRI sum score, possible values 0–12) was calculated. In each plot, the difference between sum score 2 (second evaluation) and sum score 1 (first evaluation) is plotted against the mean of the two sum scores. Dotted lines represent mean difference and 95% limits of agreement. The plots show generally smaller differences for both readers’ combined reading and for the average of both readers’ scores (average reading) than for individual reader scores. Mean difference in sum score (with 95% limits of agreement) was for reader A −0.4 (−4.4, 3.7), reader B 1.2 (−2.7, 5.1), combined reading 0.0 (−3.4, 3.4) and average reading 0.4 (−2.5, 3.3).

The MRI sum score for all ligament parts had higher intra-reader reliability with combined scoring (ICC 0.74, 95% CI: 0.64, 0.82) and both readers’ average scoring (ICC 0.78, 95% CI: 0.68, 0.84) than with individual reader scoring (A: ICC 0.55, 95% CI: 0.40, 0.67; B: ICC 0.69, 95% CI: 0.58, 0.78). SDC in MRI sum score was lowest for average scoring (2.9) followed by combined (3.3) and individual scoring (A: 4.2, B: 4.4). Similarly, MRI sum score differed less between the two evaluations when average scoring or combined scoring was used (Figure 2).

## Discussion

In this study on the reliability of two readers compared to one, the combined reading was more reproducible than a single expert’s reading. Therefore, research designs should preferentially use a combined reading for conclusive results. When reporting a sum score for several MRI findings, two readers’ average score can be used. Importantly, the two readers in our study first interpreted all images independently; then, they solved all disagreements in consensus. They did not perform a consensus reading without prior separate readings. This approach makes it impossible to assess observer variation, and it is not advised in research settings [20].

Three other points should be noted. First, the combined reading improved the reliability of results, despite the fact that the expert alone achieved moderate to good reliability. Because this level of reliability is common in diagnostic imaging [21-23], our findings may be generalized to many types of imaging examinations. Second, the additional reader had moderate experience. A second expert might have provided even more improvement in the reliability. Third, the consensus reading in cases of agreement may be useful, because consensus discussions can improve agreement between readers [2,24].

The prevalence of a high signal intensity score increased from the first to the second reading (Table 2). This was probably due to uncertainty in interpretation or due to a response bias (i.e., the readers’ tendency to prefer scoring high or low, particularly when in doubt, independently of the signal characteristics [25]). Interestingly, the prevalence of a high signal intensity score increased between evaluations less when based on both readers’ combined reading, probably because ambiguous cases were more likely to be discussed in consensus and scored consistently. In a prior study [16], each of two readers evaluated the signal intensity of the alar ligament, and assigned lower scores in the second reading than in the first.

In some previous MRI studies of the alar and transverse ligaments, conclusive ligament interpretations were based on one reader’s evaluation [26,27], two readers’ consensus evaluation (without prior separate readings) [28], or two readers’ combined reading (i.e., separate readings followed by consensus reading in cases of disagreement) [13,29,30]. Many factors affected the quality of these studies in addition to the reliability of the conclusive ligament interpretation. Nevertheless, our data indicated that the conclusive interpretation was more reproducible when based on the combined reading, compared to a single expert reading.

Combined readings were performed in our larger studies on high signal intensities of alar and transverse ligaments. In those studies, the same two readers that were used in the present study independently scored all ligaments, solved all disagreements in consensus, and reported a conclusive combined score [11,12,31,32]. Based on that conclusive score, the high intensity signal differed little between healthy volunteers and patients with rheumatoid arthritis, chronic neck pain, or acute whiplash; furthermore, the conclusive score did not affect outcome after acute whiplash [11,12,31,32]. The process of conclusive image reading had been optimized to improve the reliability, which is essential before assessing validity [1]. Neglecting this optimization might lead to underestimations of the finding’s potential relationship to clinical features, outcome, or treatment effects [33,34].

Our findings support the use of two or more readers for determining conclusive image readings in research, particularly for images with some ambiguity. In cases where a reliable result was previously documented, one expert’s reading might be sufficient for a conclusive reading. The use of two readers must be weighted against the additional effort required to solve disagreements in consensus or to employ additional readers. MRIs of upper neck ligaments yield limited clinical information, and they are not recommended for routine clinical use [12,13,31,32,35,36]. It has been speculated that the ligament high signal intensities may represent normal morphological ligament variants with loose connective tissue and/or fat [13,27,31]. Nevertheless, the present study suggested that more than one reader would provide benefit, e.g., on MRI findings in a whiplash patient. Further studies of clinically important image findings are required to confirm the higher reliability of two readers compared to one.

An important unresolved question is whether a “two readers approach” provides better agreement with a clinical “gold” standard than readout with one single expert, or has better predictive utility. No “gold” standard exists and no predictive utility has been documented for the MRI findings evaluated in this study. It is also not clear whether a “multiple readers approach” with use of more than two independent readers’ majority score may be more reliable, accurate or clinically useful, or whether an assessment of discrepant reads *per se* may provide clinically more relevant information than consensus reading in cases of disagreement.

A major strength of this study was that the readers were blinded to the study design. The study had a moderate sample size and power. However, all differences in kappa values, in *P* values for disagreement on prevalence, in ICCs, in SDC, and on Bland Altman plots were in the same direction; this indicated higher reliability with two readers than with one. The kappa for all ligament parts together indicated significantly higher reliability based on non-overlapping CIs. These CIs assumed independent scoring of the four ligament parts; however, all four parts were visible on the same image. Thus, some dependency in the scoring probably existed and may have narrowed the CIs. The kappa value is affected by the prevalence of the evaluated finding, and it is difficult to compare between groups that differ in prevalence [19]. However, this effect on kappa is largest for prevalence below 10% and above 90% and smaller for the prevalence reported in our study (22.5% - 46.1%, Table 2). Normality plots suggested only small deviations from the assumed normal distribution and the ICCs were also higher with two readers than with one based on log transformed data. Our study included more healthy subjects than patients. Images from a sample with a higher proportion of patients would be likely to show a similar prevalence of high signal intensities in the ligaments (based on findings in our main project). However, those images might have been more difficult to interpret, which might have led to lower reliability, and ultimately, a larger improvement in reliability with the inclusion of a second reader. Therefore, this limitation tends to strengthen our findings.

## Conclusions

To our knowledge, this study was the first to provide empirical data on the reliability of two readers compared to one. For scoring the signal intensities of ligaments on upper neck MRIs, image reading by a single expert was less consistent than combined reading by the same expert in collaboration with a second reader. The latter approach implied independent readings followed by consensus reading in cases of disagreement. This approach was used to determine conclusive interpretations of high intensity signals in neck ligament studies that have previously shown that high signal intensities had limited clinical relevance [11-13,31,32,36]. Two or more readers may be needed to provide reliable conclusive image reading results in research. In this study, a moderately experienced second reader improved the reliability compared to a single expert reader. The implications of this for clinical work should be assessed in further studies of more clinically relevant imaging findings.

## Competing interests

The authors declare that they have no competing interests.

## Authors’ contributions

AE conceived, designed, and coordinated the study, performed statistical analyses, and drafted the manuscript. NV interpreted the MRI examinations, performed statistical analyses, and helped draft the manuscript. JK interpreted the MRI examinations, participated in the data analysis, and reviewed the manuscript. All authors read and approved the final manuscript.

## Pre-publication history

The pre-publication history for this paper can be accessed here:

http://www.biomedcentral.com/1471-2342/13/4/prepub

## Supplementary Material

Additional file 1 Hypothetical example of a case in which two readers combined provided less consistent scores than that provided by either reader individually. Click here for file
