# Supplementary material for: Are two readers more reliable than one? A study of upper neck ligament scoring on magnetic resonance images
Source: BMC Med Imaging. 2013 Jan 17;13:4. doi: 10.1186/1471-2342-13-4 (PMC3626747; doi:10.1186/1471-2342-13-4)
Supplement: Additional file 1 — Hypothetical example of a case in which two readers combined provided less consistent scores than that provided by either reader individually. [file 1471-2342-13-4-S1.doc]

| **Additional file 1 – Hypothetical example of a case in which two readers combined provided less consistent scores than that provided by either reader individually** | | | | | | | | | | | |
| --- | --- | --- | --- | --- | --- | --- | --- | --- | --- | --- | --- |
|  | | Subject number | | | | | | | | | |
|  | | 1 | 2 | 3 | 4 | 5 | 6 | 7 | 8 | 9 | 10 |
| Initial evaluation | | | | | | | | | | | |
|  | Reader A | 1 | 1 | 1 | 0 | 0 | 0 | 0 | 1 | 1 | 1 |
|  | Reader B | 1 | 1 | 1 | 0 | 0 | 1 | 1 | 0 | 0 | 0 |
|  | A and B score after consensus | 1 | 1 | 1 | 0 | 0 | 0 | 0 | 1 | 1 | 0 |
| Second evaluation (**bold** scores are different from those in the initial evaluation) | | | | | | | | | | | |
|  | Reader A | 1 | 1 | 1 | 0 | **1** | 0 | 0 | 1 | 1 | **0** |
|  | Reader B | 1 | 1 | 1 | 0 | 0 | **0** | 1 | 0 | 0 | **1** |
|  | A and B score after consensus | 1 | 1 | 1 | 0 | **1** | 0 | **1** | **0** | **0** | 0 |
| The table shows hypothetical data of scores from two readers, A and B. The readers were assumed to be blinded for evaluations and re-evaluations of the same images after a period of 3 months. A finding of 0 (not present) or 1 (present) was assigned to images from 10 subjects. The table shows potential results when readers A and B first independently scored all cases and then resolved all disagreements in consensus and reported a final score. The prevalence of score 1 was the same in the initial and second evaluations: 60% for A, 50% for B, and 50% for A and B after consensus. However, the agreement between the initial and second evaluations was lower for readers A and B after consensus (kappa 0.20) than for each reader individually (kappa 0.58 for A and 0.60 for B). | | | | | | | | | | | |
